# School climate and school identification as determinants of smoking conventional cigarettes or vaping among adolescents in China: Stress-coping mediation mechanisms

**DOI:** 10.18332/tid/177171

**Published:** 2024-02-15

**Authors:** Yanqiu Yu, Mengni Du, Deborah Baofeng Wang, Anise M. S. Wu, Juliet H. Chen, Siman Li, Stefanie H. Y. Yen, Guohua Zhang, Dajin Du, Mingxuan Du, Joseph T. F. Lau

**Affiliations:** 1 School of Public Health, Fudan University, Shanghai, China; 2 Teaching and Research Center, Bureau of Education, Linhai, China; 3 Zhejiang Provincial Clinical Research Center for Mental Disorders, The Affiliated Wenzhou Kangning Hospital, Wenzhou Medical University, Wenzhou, China; 4 Department of Psychology, Faculty of Social Sciences, University of Macau, Macau, China; 5 Centre for Cognitive and Brain Sciences, Institute of Collaborative Innovation, University of Macau, Macau, China; 6 Department of Psychology, Zhejiang Sci-Tech University, Hangzhou, China; 7 Center for Health Behaviors Research, Jockey Club School of Public Health and Primary Care, The Chinese University of Hong Kong, Hong Kong SAR, China; 8 School of Mental Health, Wenzhou Medical University, Wenzhou, China; 9 Public Mental Health Center, School of Mental Health, Wenzhou Medical University, Wenzhou, China

**Keywords:** Stress Coping Theory, school climate, school identification, smoking, adolescents, vaping

## Abstract

**INTRODUCTION:**

Smoking conventional cigarettes or vaping (SV) poses significant health threats to adolescents. School climate and school identification are key elements of the school environment and potential factors of SV. Based on the Stress Coping Theory, the mediations between school climate/school identification and SV, via perceived stress/active coping, were examined.

**METHODS:**

A cross-sectional survey was conducted among secondary school students from February to March 2022 in Taizhou, China. Structural equation modeling was used.

**RESULTS:**

The prevalence of SV among the 7526 participants was 4.7% (singular use of conventional cigarettes: 3.2%; singular use of electronic cigarettes: 3.6%; dual use: 2.1%). School climate, school identification, and active coping were positively, and perceived stress (family stress, academic stress, and peer-related stress) were negatively associated with SV. The association between school climate and SV was fully mediated via: 1) school climate → perceived stress → SV; 2) school climate → active coping → SV; and 3) school climate → perceived stress → active coping → SV. The effect sizes were 52.1%, 43.8%, and 6.3%, respectively. Similar partial mediation mechanisms were found between school identification and SV, with relatively small effect sizes (<10%).

**CONCLUSIONS:**

This study observed the prevalence of SV among Chinese secondary school students. School climate and school identification had both significant direct and indirect (via perceived stress/active coping) effects on SV. Positive school environments may reduce students’ stress and promote active coping. The stress coping mechanisms explained the association between school climate and SV better than between school identification and SV.

## INTRODUCTION

Adolescent smoking is of great public health significance, as it may last throughout adulthood^1^. The onset of smoking before adulthood was associated with a lower likelihood of quitting smoking, a greater risk of nicotine dependence, and physical and psychological illness^2-4^. Regarding smoking conventional cigarettes, a national survey conducted in 31 Chinese provinces reported a prevalence of ever smoking and current smoking of 16.7% and 4.7% among adolescents in 2021, respectively^5^. Globally, the use of electronic cigarettes (i.e. vaping) may have become a trend and an emerging health threat. The harms of vaping are widely reported (e.g. health risks and addiction propensity), although there are benefits (e.g. harm reduction and reduced exposure to secondhand smoke)^6^. Despite mixed evidence, there are also worries that vaping would lead to conventional smoking^6^. The aforementioned report documented that 16.1% and 3.6% of Chinese middle school students had ever and currently vaped in 2021, versus 3.5% and 0.8% in 2019, respectively^5^. To inform effective interventions, research about factors of smoking conventional cigarettes or vaping (SV) and related mechanisms among Chinese adolescents is warranted.

The socioecological model postulates that the environment is one of the key determinants of individual health-related behaviors^7^. Hence, it was expected that the school environment would affect adolescent risk behaviors (e.g. SV). School climate and school identification are two key intercorrelated, yet different components of the social environment. The former relates to interpersonal relationships, academic challenges, and shared culture/values in the school setting^8^; the latter reflects a sense of belonging to the school^9^. In the literature, a longitudinal study reported that a positive school climate was negatively associated with substance use (including SV) in American adolescents^10^. Lower school attachment, a construct closely related to school identification^11^, was associated with a higher risk of conventional smoking^12^. Although some variables related to social environments (e.g. school safety and school satisfaction^13,14^) were associated with adolescent conventional smoking, to our knowledge, no studies have inspected the associations between school climate/school identification and vaping or the mediation mechanisms of such associations.

The Lazarus and Folkman^15^ transactional theory of stress and coping was used as the theoretical framework of this study to investigate the mediations between school climate/school identification and SV. The theory postulates that environmental stimuli seen as threatening, challenging, or harmful would become stressors and result in stress and/or distress. Such would induce various coping strategies (e.g. active coping), which may in turn lead to favorable/unfavorable coping outcomes/responses (e.g. SV)^15,16^. The stress-coping theory has been commonly applied to understanding factors and mechanisms involving addictive behaviors, including smoking^17^. Poor school climate and low school-identification can be interpreted as stressors that would increase perceived stress. The stressors and resultant perceived stress may reduce the use of active coping, which may, in turn, increase maladaptive coping responses involving SV. The associations between school climate and perceived stress/active coping^12-14,18,19^ and between perceived stress/active coping and SV^20,21^, have gained empirical support.

The present study investigated the prevalence of SV among adolescents in a Chinese city. First, it tested the significance of four factors of SV, including school climate, school identification, perceived stress, and active coping. Second, it was hypothesized that the association between school climate and SV would be mediated via: 1) two 2-step paths of school climate → perceived stress → SV and school climate → active coping → SV; and 2) a 3-step path of school climate → perceived stress → active coping → SV. Third, similar mediation hypotheses were tested, replacing the independent variable of school climate with school identification.

## METHODS

### Participants and data collection

A cross-sectional survey was conducted among students from five junior middle schools (generally representing ages 12–15 years), three senior middle schools (generally representing ages 15–18 years), and one vocational secondary school (generally representing ages 15–18 years), from 20 February to 4 March 2022, in Taizhou, China^22^. With the assistance of the local education sector, the above participating schools were conveniently selected; the principals consented to the schools’ participation. All students of the first two grades of the selected schools were invited to participate in this study. Before the commencement of the survey, schoolteachers informed the parents about the objective and content of this survey via Wechat groups that were used for teacher–parent communication; students were also requested to inform their parents in person; parental opt-out options were provided in both channels, but no opt-out form was received. In the classroom setting and with the absence of schoolteachers, the well-trained fieldworkers verbally briefed the students on the logistic and anonymous nature of the survey. Furthermore, the voluntary nature of participation was emphasized to the students that they had the right to quit the survey at any time without any negative consequences and that the submission of the completed questionnaire would indicate their willingness to join the survey. After the verbal briefing and confirmation of the student’s full understanding, the fieldworkers distributed the questionnaires with a cover page covering all the above briefing information. The questionnaire took about half an hour to complete, and the fieldworkers were present for inquiries. Those who were unwilling to participate were arranged to stay in a separate classroom to read or self-study. No incentive was given to the students, parents, and schoolteachers. The ethics committee of the corresponding author’s affiliated institution approved the study procedure and questionnaire.

A total of 8285 students returned their completed questionnaires, of which 114 (1.4%) were excluded as more than 20% of all the item responses had missing data, and 615 (7.4%) were excluded as their responses failed to pass the built-in logic checks. In addition, 30 (0.4%) questionnaires were removed due to missing data in the questions regarding SV. The final sample size for data analysis was 7526 (99.6%).

### Measures

*Background information*

Information was collected, including age, school type (junior middle school, senior middle school, and vocational secondary school), sex, whether being a Taizhou resident, whether living with both parents, and the education level of the father and mother.

*Smoking conventional cigarettes or vaping (SV)*

An item asked whether the participant had smoked conventional cigarettes in the past 12 months (yes/no). Another item asked whether the participants had vaped in the past 12 months (yes/no). A composite index of SV was generated for those who had smoked conventional cigarettes or vaped. It was used as the binary dependent variable of this study.

### School climate and school identification

This was assessed using the 15-item Abbreviated Version of the Dual School Climate and School Identification Measure – Student (SCASIM-St15), validated in Chinese adolescents with satisfactory psychometric properties^22^. This scale comprises four first-order factors of school climate (i.e. student-student relations, staff-student relations, academic emphasis, and shared values and approach) and an additional fifth factor assessed school identification^22^. Sample items were: ‘Students are fair to each other’ for student-student relations, ‘Staff are fair in their dealing with students’ for staff-student relations, ‘Teachers want every student to do their best’ for academic emphasis, ‘There is school spirit and pride’ for shared values and approach, and ‘I feel a strong connection with this school’ for school identification (1=strongly disagree to 5=strongly agree). The Cronbach’s alpha of the five subscales ranged from 0.89 to 0.96 in this study.

### Perceived stress

A 3-item scale was used. The items were: ‘To what extent do you perceive stress generated from family/academic situation/peer relationship (0=none at all to 10=extremely high)?’ The Cronbach’s alpha of this scale was 0.68 in this study.

### Active coping

This was assessed using the two-item active coping subscale of the Brief COPE questionnaire; its Chinese version has been validated with satisfactory psychometric properties^23^. A sample item was: ‘I've been taking action to try to improve the situation’ (1=never to 5=always). The Cronbach’s alpha of the subscale was 0.88 in this study.

### Statistical analysis

The kappa coefficient was generated to test the level of consistency/overlap between those smoking conventional cigarettes and vaping. Spearman correlation coefficients were generated to test the correlations among the potential factors of SV. Univariate and multivariable logistic regression analyses adjusting for background factors were conducted to test the significance and directions of the associations between factors and SV. Structural equation modelling (SEM)^24^ was performed to test the potential mediation mechanisms. One advantage of the SEM is its involvement of both observed and unobserved (latent) variables; an observed variable refers to a variable that could be directly observed or measured while a latent variable represents underlying constructs or concepts that are not directly measurable. Latent variables are often generated from item/scale scores to represent underlying constructs and reduce scale assessment measurement errors. In this study, four latent variables were generated. The latent variable of school climate was generated from the four subscales of school climate of the SCASIM-St15. In contrast, the other three latent variables of school identification, perceived stress, and active coping, were generated from the corresponding original items. The mediation effects of perceived stress and active coping (mediator) between school climate/school identification (the independent variable, IV) and SV (the dependent variable, DV) were examined, after adjusting for background factors to remove potential confounding effects. In the mediation, the direct effect refers to the path from the IV to the DV (e.g. school climate → SV) while the indirect effect involves a path from the IV to the mediator together with a path from the mediator to the DV (e.g. school climate → perceived stress → SV), indicating that the mediator might account for the association between the IV and the DV. The effect size of the mediation effect was calculated by dividing the indirect effect by the total effect (i.e. the addition of both direct and indirect effects). Standardized coefficients (β) were reported in SEM. As SV was a binary DV, the Weighted Least Square Mean and Variance Adjusted estimator was used in SEM. Goodness-of-fit of the SEM would be satisfactory when the Comparative Fit Index (CFI) and Tucker-Lewis Index were ≥0.90 and the Root Mean Square Error of Approximation (RMSEA) was ≤0.08^24^. To provide supplementary information, the same data analyses were conducted using the two dependent variables of smoking conventional cigarettes and vaping separately in two sets of models. SEM was conducted using Mplus 7.0, while the other tests were analyzed using SPSS 23.0. Statistical significance was defined as p<0.05 (two-tailed tests).

## RESULTS

### Descriptive statistics

The mean age of the participants was 14.9 years (SD=1.5; range: 10–20); 62.5%, 30.7%, and 6.8% were junior middle school, senior middle school, and vocational secondary school students, respectively. Over half were male (54.3%); 16.6% were not Taizhou residents; and over a quarter were not living with both parents (26.0%). The father (10.1%) and mother (10.4%) of about 10% of the participants had an education level of college or above. The prevalence of the singular use of conventional cigarettes and e-cigarettes in the past 12 months was 3.2% and 3.6%, respectively. The prevalence of SV was 4.7% (Table 1). The prevalence of the dual use of conventional smoking and vaping was 2.1%, and the kappa value between them was 0.60 (p<0.001). The mean (SD) and range of the scores of the independent variables and the potential mediators are presented in Table 2.

**Table 1:** Participant characteristics within the cross-sectional survey conducted among secondary school students, February to March 2022, in Taizhou, China (N=7526)

*Characteristics*	*All n (column %)*	*Smoking conventional cigarettes or vaping*	
		*n (row %)*	*OR (95% CI)*
**Total**	7526 (100)	357 (4.7)	
**Age** (years)			
**School type**			1.24 (1.16–1.33)***
Junior middle school ®	4700 (62.5)	176 (3.7)	1
Senior middle school	2307 (30.7)	148 (6.4)	1.78 (1.41–2.21)***
Vocational secondary school	519 (6.8)	33 (6.4)	1.75 (1.19–2.56)***
**Gender**			
Female ®	3384 (45.0)	94 (2.8)	1
Male	4090 (54.3)	263 (6.4)	2.41 (1.89–3.06)***
Missing data	52 (0.7)	0 (0.0)	
**Taizhou residents**			
Yes ®	6212 (82.5)	291 (4.7)	1
No	1247 (16.6)	60 (4.8)	1.03 (0.77–1.37)
Missing data	67 (0.9)	6 (9.0)	
**Living with both parents**			
Yes ®	5533 (73.5)	227 (4.1)	1
No	1955 (26.0)	129 (6.6)	1.65 (1.32–2.06)***
Missing data	38 (0.5)	1 (2.6)	
**Father’s education level**			
Junior middle school or lower ®	4822 (64.1)	224 (4.6)	1
Senior middle school/vocational high school	1751 (23.3)	100 (5.7)	1.24 (0.98–1.58)
College or higher	760 (10.1)	24 (3.2)	0.67 (0.44–1.03)
Missing data	193 (2.6)	9 (4.7)	
**Mother’s education level**			
Junior middle school or lower ®	5105 (67.8)	242 (4.7)	1
Senior middle school/vocational high school	1406 (18.7)	68 (4.8)	1.02 (0.78–1.35)
College or higher	779 (10.4)	33 (5.9)	0.89 (0.61–1.29)
Missing data	236 (3.1)	14 (5.9)	
**Smoking conventional cigarettes**			
No	7282 (96.8)		
Yes	244 (3.2)		
**Vaping**			
No	7256 (96.4)		
Yes	270 (3.6)		

Missing data were excluded from the logistic regression analysis. Structural equation modeling was used. ® Reference categories.

*** p<0.001.

**Table 2:** Descriptive statistics and correlation matrix, among secondary school students, February to March 2022, in Taizhou, China

	*Range*	*Mean (SD)*	*1*	*2*	*3*	*4*	*5*	*6*	*7*	*8*
**School climate**										
1. Student-student relationship	3–15	11.8 (3.1)	-							
2. Student-staff relations	3–15	11.9 (2.9)	0.58***	-						
3. Academic emphasis	3–15	11.9 (2.8)	0.54***	0.84***	-					
4. Shared-values approach	3–15	11.3 (3.2)	0.60***	0.77***	0.80***	-				
5. School identification	3–15	11.2 (3.3)	0.57***	0.72***	0.73***	0.86***	-			
**Perceived stress**										
6. Family-related stress	0–10	3.2 (2.8)	-0.22***	-0.22***	-0.21***	-0.25***	-0.26***	-		
7. Academic stress	0–10	5.6 (2.9)	-0.20***	-0.23***	-0.20***	-0.25***	-0.26***	0.44***	-	
8. Peer stress	0–10	2.6 (2.7)	-0.25***	-0.23***	-0.23***	-0.26***	-0.25***	0.47***	0.35***	-
9. Active coping	0–21	5.7 (1.6)	0.28***	0.32***	0.32***	0.35***	0.37***	-0.18***	-0.14***	-0.18***

SD: standard deviation.

*** p<0.001.

### Background factors of SV

In Table 1, older age (OR=1.24; 95% CI: 1.16–1.33), senior middle school students versus junior middle school students (OR=1.78; 95% CI: 1.41–2.21), and vocational secondary school students versus junior middle school students (OR=1.75; 95% CI: 1.19–2.56), male sex (OR=2.41; 95% CI: 1.89–3.06), and not living with both parents (OR=1.65; 95% CI: 1.32–2.06), were positively associated with SV. The other associations between Taizhou residence/father’s education level/mother’s education level and SV were statistically not significant.

### Associations between the independent variables/mediators and SV

Consistent with the results of the univariate logistic regression analysis, the results of the multivariable logistic regression analysis (adjusted for background factors) showed that the four subscales of school climate, the school identification scale, and active coping (AOR: 0.78–0.92) were all negatively and significantly associated with SV. The three types of perceived stress involving family, academic domain, and peer relationship (AOR: 1.07–1.14) were positively associated with SV (Table 3).

**Table 3:** Factors of smoking conventional cigarettes or vaping among secondary school students, February to March 2022, in Taizhou, China (N=7526)

*Factors*	*OR (95% CI)*	*AOR (95% CI)*
**School climate**		
Student-student relationship	0.92 (0.89–0.95)***	0.92 (0.89–0.95)***
Student-staff relations	0.86 (0.83–0.89)***	0.87 (0.84–0.90)***
Academic emphasis	0.86 (0.83–0.89)***	0.87 (0.84–0.91)***
Shared-values approach	0.87 (0.84–0.90)***	0.89 (0.86–0.92)***
**School identification**	0.85 (0.82–0.88)***	0.86 (0.83–0.89)***
**Perceived stress**		
Family-related stress	1.15 (1.11–1.19)***	1.14 (1.10–1.19)***
Academic stress	1.09 (1.04–1.13)***	1.09 (1.04–1.13)***
Peer stress	1.07 (1.03–1.11)**	1.07 (1.03–1.12)**
**Active coping**	0.79 (0.73–0.84)***	0.78 (0.72–0.84)***

AOR: adjusted odds ratio; the models were adjusted for background factors, including age, school type, gender, whether living in the studied city constantly, whether living with both parents, and the education level of the father and mother.

** p<0.01,

*** p<0.001.

### Correlations

The four subscales of school climate and the school identification subscale were negatively correlated with the three types of perceived stress (r: -0.26 – -0.20); all of them were positively correlated with active coping (r: 0.28–0.37). The three types of perceived stress were all negatively correlated with active coping (r: -0.18 – -0.14). The four subscales of school climate and the school identification subscale were positively correlated with each other (r: 0.54–0.86). Similarly, significant positive correlations among the three types of perceived stress were observed (r: 0.35–0.47) (Table 2).

### SEM testing the mediation mechanisms

The results of the confirmatory factor analysis (Supplementary file Figure S1) for the four latent variables showed satisfactory model fit indices (CFI=0.96; TLI=0.94; RMSEA=0.07). The factor loadings of the latent variables ranged 0.61–0.95 (all p<0.001). The results indicate that the latent variables were suitable for conducting SEM.

**Figure 1:**
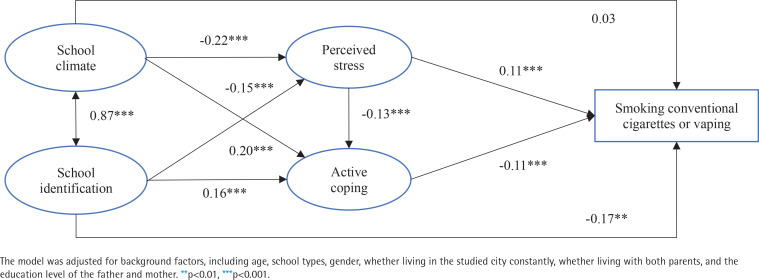
Structural equation modeling. Rectangles represent observed variables, ovals represent latent variables, arrows represent the directions of the associations, and straight lines with coefficients represent the standardized path coefficients

Figure 1 presents the SEM model, which showed satisfactory model fit indices (CFI=0.97; TLI=0.96; RMSEA=0.02). The results revealed two significant 2-step paths and one significant 3-step path between school climate and SV. The significant mediation via perceived stress (β= -0.03; p<0.001; effect size=52.1%) indicated that school climate was positively associated with perceived stress (β= -0.22; p<0.001), which was in turn positively associated with SV (β=0.11, p<0.001). The significant mediation via active coping (β= -0.02; p=0.001; effect size=43.8%) indicated that school climate was positively associated with active coping (β=0.20; p<0.001), which in turn was negatively associated with SV (β= -0.11; p<0.001). In the 3-step mediation, the association between school climate and SV was firstly via perceived stress and then via active coping (β= -0.01; p=0.002; effect size= 6.3%). All the corresponding two 2-step paths and the 3-step path between school identification and SV were also of statistical significance; the effect size of such three mediation paths involving school identification was 8.2% (via perceived stress only; β= -0.02; p=0.004), 8.2% (via active coping only; β= -0.02; p=0.001), and 1.0% (via perceived stress and then active coping; β= -0.01; p=0.005), respectively. Furthermore, full mediation was found between school climate and SV via perceived stress/active coping, as the direct effect from school climate to SV was statistically not significant (β=0.03; p=0.646). In contrast, the mediation between school identification and SV was partial, as the direct path was statistically significant (β= -0.17; p=0.007).

Multi-group SEM was further conducted to test whether the above mediation model would be moderated by sex. The results showed that all the structural and indirect paths were invariant in males and females (Supplementary file Table S1), indicating that the above mechanisms applied to both sexes.

### Supplementary analyses using smoking conversational cigarettes and vaping as two separate dependent variables

The logistic regression analyses and SEM were repeated by using smoking conventional cigarettes and vaping as two separate dependent variables, respectively. The results are presented in Supplementary file Table S2 and Figures S2 and S3. The significance and directions of all the associations and mediation mechanisms were consistent with those using SV as a combined dependent variable.

## DISCUSSION

The observed prevalence of the singular use of conventional/electronic cigarettes among Chinese adolescents was 3.2/3.6%, which was comparable with the national figures reported in China in 2019 (4.7/3.6%)^5^ and in England, Scotland, and Wales (4.9–11.0/2–3%)^25,26^, but was higher than the Japanese prevalence (0.6/0.7%)^27^. In the present study, the prevalence of SV was 4.7%, denoting a moderate but not total overlap as seen by the significant kappa statistics. Given the large population size of 31 million secondary students in China in 2021 and the negative short-term/long-term consequences due to smoking/vaping, the potentially large absolute number of adolescent smokers/vapers suggests the need for interventions in reducing adolescent SV. Extra attention should be paid to males and students of senior middle schools and vocational secondary schools, as they might receive less parental supervision and exhibit more behavioral problems. Disadvantaged students (e.g. those not living with parents) who showed higher smoking prevalence than others, in this and previous studies^28^, also demand special attention.

Corroborating the extant literature^10^, this study found that school climate was positively associated with SV and newly added to the literature that school identification was negatively associated with SV. Such findings underline the importance of improving school-related environments, as the socioecological model postulates^7^. According to the World Health Organization Health Promoting Schools framework, a positive school climate can be created by strengthening students’ social and problem-solving skills, engaging them in decision-making processes in the school community, and providing them with knowledge about health/risk behaviors^29^. It is also important to cultivate a sense of school belonging to strengthen school identification^9^ which can be achieved by encouraging in-school social interactions, embracing diversity, and establishing clear expectations and guidelines regarding bullying, harassment, and discrimination^30^. A cluster randomized trial in the UK reported the effectiveness of a whole-school environment intervention on students’ psychological and behavioral outcomes including smoking; the intervention components included staff training in restorative practice, establishing school action groups, and student training on social and emotional skills^31^.

Based on the framework of the transactional theory of stress and coping^15^, this study revealed the mediation mechanisms between school climate/school identification and SV, via perceived stress and active coping (i.e. two 2-step and one 3-step indirect effects). The results were consistent with the relationships mentioned in the Introduction^12-14,18-21^. Notably, the association between school climate and SV, was fully mediated by the tested indirect paths; both 2-step paths via perceived stress and active coping showed a large mediation effect size of over 40% while the 3-step path took up about 6% of the variance. Thus, improvements in school climate may reduce stress and improve active coping, which may largely reduce students’ SV. In contrast, the effect size of the three indirect paths mediating between school identification and SV, was partial and relatively small (about 8%), suggesting the plausible existence of other uninvestigated mediators such as self-image which was associated with both school environment and SV^32^. Notably, school climate and identification were moderately to strongly correlated with each other, and both were significantly associated with SV. Thus, the above results should not be interpreted as a suggestion that school identification was less important than school climate in affecting adolescent SV, but instead, the two associations between school climate/school identification and SV, might involve different mechanisms. In other words, the stress-coping mechanism may be more applicable to the association between school climate and SV, than that between school identification and SV, when both of them were considered simultaneously in the same model.

## Implications

The findings have practical implications for health promotion. The significant stress-coping mechanism highlights that it is important to reduce perceived stress and promote active coping among adolescents by improving school climate. In addition, middle school students may face many stressors in terms of academic, interpersonal (e.g. peer and family relationships), and developmental (e.g. puberty change) issues^33^. Skill training on stress management should be emphasized, as stress was associated with maladaptive outcomes involving risky behaviors^16^. A brief stress mindset intervention may be an effective and innovative approach; it aims to shift individuals’ mindsets from stress-is-debilitating to stress-is-enhancing^34^. Furthermore, providing social support is effective for stress management and improving active coping^35^.

### Strengths and limitations

This study has the strength of identifying some novel factors of SV (school climate and school identification) and related mechanisms. However, there are several limitations. First, this study precludes causal or temporal inference due to the cross-sectional design. Second, the participating schools were conveniently selected from one Chinese city; generalization of the results to the entire city, other regions, or populations in China should be made cautiously. Nonetheless, as the prevalence of smoking conventional cigarettes and vaping was comparable to that of the national data, it is believed that some relevant common patterns and related mechanisms might exist among adolescents in China that allow for generalization of the findings. Confirmation is required in future studies. Third, recall bias might occur as the questionnaires were self-administered. Fourth, there might be reporting bias; SV was self-reported and not subjected to validation. As smoking and vaping may not be acceptable to teachers and parents; the prevalence of SV might have been underestimated. We asked about SV in the past 12 months and the timeframe was arbitrary. Fifth, only one type of positive coping strategy was studied, other coping strategies, including negative coping strategies (e.g. behavioral disengagement), are also highly relevant and need to be included in future studies. Sixth, perceived stress only focused on those related to family, academic situations, and peer relationships; other sources of stress (e.g. financial and health) were not included in the scale. In addition, other potential mediators (e.g. self-image) between school identification and SV were not investigated in this study. Last, there are other school-related indicators such as school belonging and school connectedness; the former refers to the extent to which the students feel accepted, supported, and included in the school setting^30^ while the latter refers to the students’ feeling of engagement and being valued in school^36^. Their concepts, compared with school climate and school identification, have similar elements yet different focuses. Future studies are warranted to explore whether there are differential effects of various school-related indicators on adolescents’ physical, mental, and behavioral health.

## CONCLUSIONS

This study evaluated the prevalence of adolescent conventional smoking and vaping in a Chinese city. Positive school climate and school identification were found to be negatively associated with adolescent SV. The significant mediation effects suggest that improvements in these two constructs (in particular school climate) may reduce perceived stress and improve active coping, and hence reduce SV, as perceived stress and active coping were also significantly associated with SV. This study has some theoretical relevance. In general, the stress coping mechanisms were able to explain the relationships between school climate/school identification and SV, but the explanation was better for the association between school climate and SV than that between school identification and SV. Future longitudinal studies and school-based interventions are warranted to verify the findings.

## Data Availability

The data supporting this research are available from the authors on reasonable request.
