# Supplementary material for: School climate and school identification as determinants of smoking conventional cigarettes or vaping among adolescents in China: Stress-coping mediation mechanisms
Source: Tob Induc Dis. 2024 Feb 15;22:10.18332/tid/177171. doi: 10.18332/tid/177171 (PMC10867738; doi:10.18332/tid/177171)
Supplement: Supplementary file 1 [file TID-22-39-s1.pdf]

Table S1 Multiple-group structural equation modeling by sex

	Male		Female		Wald test		
	β	*p*	*β*	*p*	Estimate	*p*	Invariance
Structural path							
School climate → Perceived stress	-0.28	<0.001	-0.27	<0.001	0.01	0.906	Supported
School climate → Active coping	0.37	<0.001	0.36	<0.001	0.14	0.711	Supported
School climate → SV	-0.18	<0.001	-0.19	<0.001	0.08	0.780	Supported
School identification → Perceived stress	-0.15	<0.001	-0.15	<0.001	0.01	0.906	Supported
School identification → Active coping	0.20	<0.001	0.19	<0.001	0.14	0.711	Supported
School identification → SV	-0.10	<0.001	-0.10	<0.001	0.08	0.780	Supported
Perceived stress → Active coping	0.22	<0.001	0.22	<0.001	0.14	0.711	Supported
Perceived stress → SV	-0.15	<0.001	-0.17	<0.001	0.08	0.780	Supported
Active coping → SV	-0.07	<0.001	-0.07	<0.001	0.08	0.780	Supported
Indirect path							
School climate → Perceived stress → SV	0.02	<0.001	0.03	<0.001	0.10	0.757	Supported
School climate → Active coping → SV	-0.02	<0.001	-0.03	<0.001	0.12	0.734	Supported
School climate → Perceived stress → Active coping → SV	0.01	<0.001	0.01	<0.001	0.13	0.718	Supported
School identification → Perceived stress → SV	0.02	<0.001	0.03	<0.001	0.10	0.757	Supported
School identification → Active coping → SV	-0.01	<0.001	-0.01	<0.001	0.12	0.734	Supported
School identification → Perceived stress → Active coping → SV	0.01	<0.001	0.01	<0.001	0.13	0.718	Supported

Note. SV = Smoking conventional cigarettes or vaping; *P* value <0.05 in Wald test denotes a significant moderation effect of sex on the tested path.

Table S2 Adjusted associations between key variables and adolescent smoking conventional cigarettes/vaping

	Smoking conventional cigarettes	Vaping
	ORa (95% CI)	ORa (95% CI)
**School climate**		
Student-student relationship	0.89 (0.86, 0.93)***	0.95 (0.91, 0.99)**
Student-staff relations	0.87 (0.84, 0.91)***	0.86 (0.83, 0.90)***
Academic emphasis	0.87 (0.84, 0.91)***	0.87 (0.83, 0.90)***
Shared values approach	0.88 (0.85, 0.92)***	0.90 (0.86, 0.93)***
**School identification**	0.86 (0.83, 0.89)***	0.88 (0.85, 0.91)***
**Perceived stress**		
Family-related stress	1.13 (1.08, 1.18)***	1.12 (1.08, 1.17)***
Academic stress	1.05 (1.00, 1.10)*	1.07 (1.02, 1.12)***
Peer stress	1.07 (1.02, 1.12)**	1.06 (1.02, 1.11)**
**Active coping**	0.77 (0.71, 0.84)***	0.79 (0.73, 0.86)***

Note. ***, $p<0.001$. ORa = Adjusted odds ratio; CI = Confidence interval. The models were adjusted for background factors, including age, school types, gender, whether living in the studied city constantly, whether living with both parents, and father's and mother's educational level.

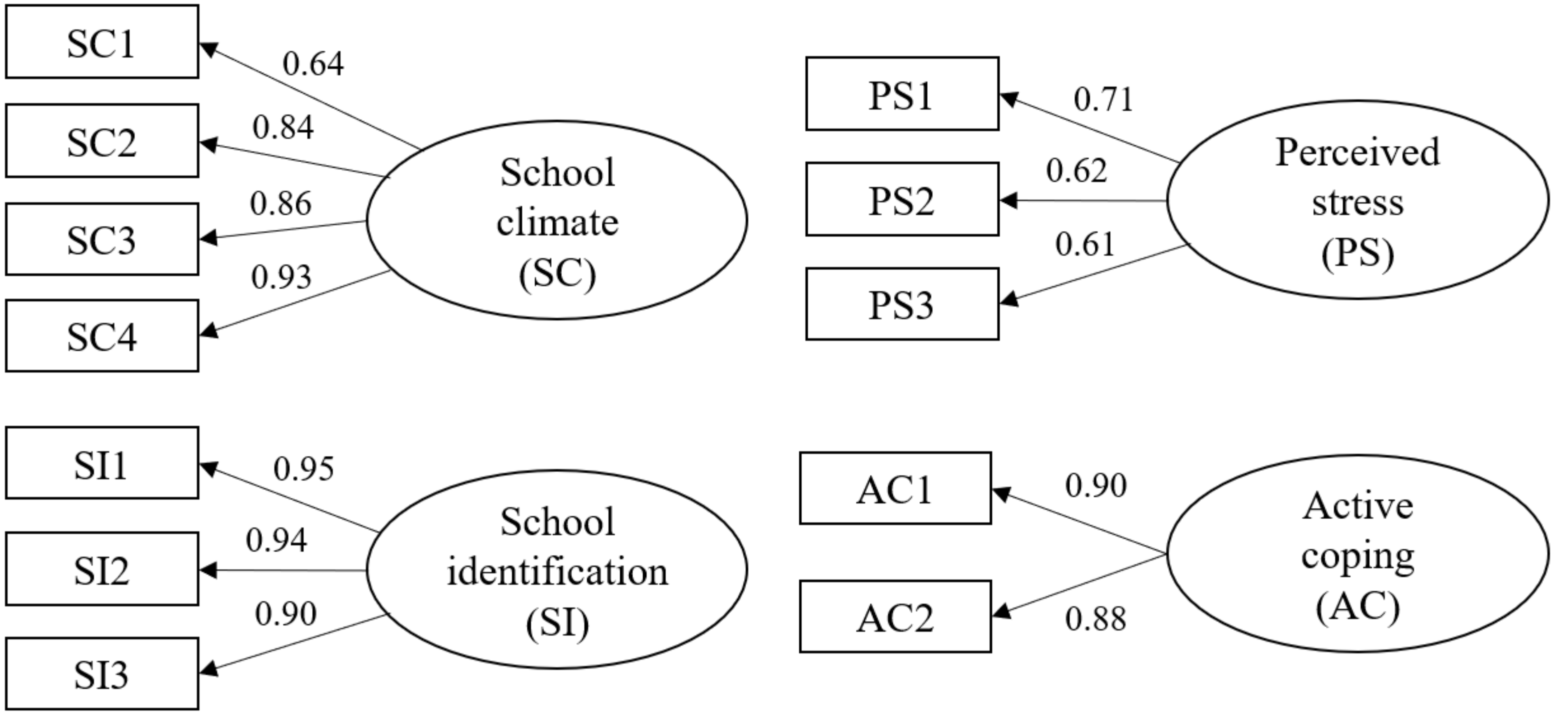

Figure S1 Confirmatory factor analysis of the latent variables (Standardized coefficients were reported. All the factor loadings were statistically significant with $p<0.001$)

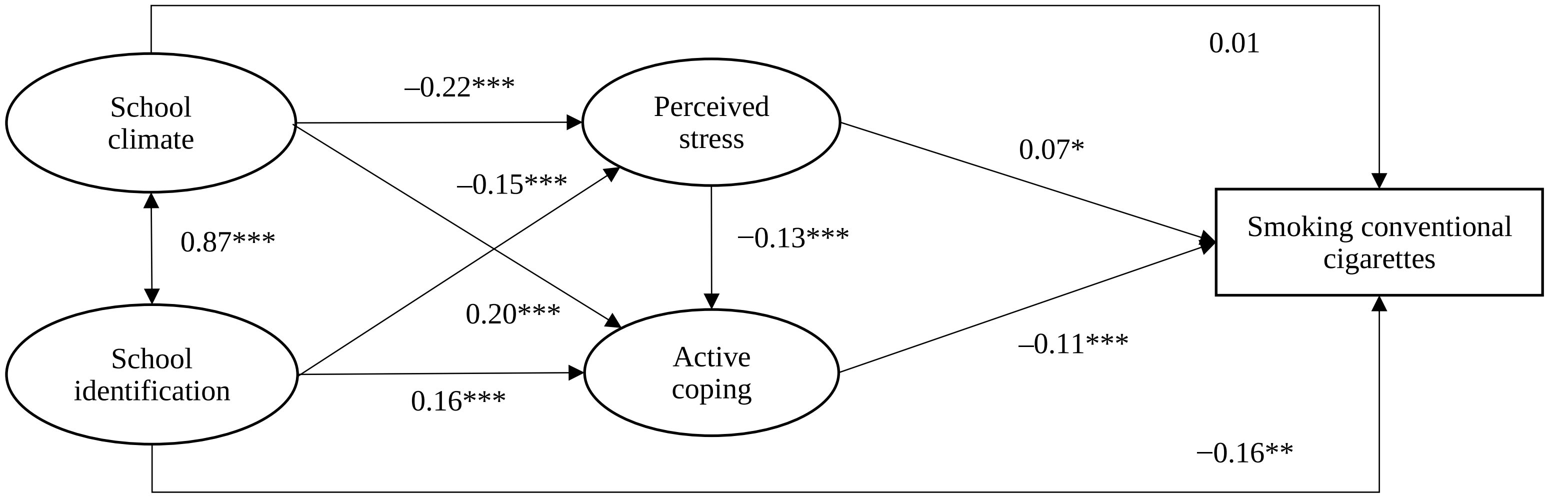

Figure S2 Structural equation modeling (**, *p*<0.01; ***, *p*<0.001. Rectangles represent observed variables, ovals represent latent variables, arrows represent the directions of the associations, and straight lines with coefficients represent the standardized path coefficients. The model was adjusted for background factors, including age, school types, gender, whether living in the studied city constantly, whether living with both parents, and father's and mother's educational level. Model fit indices: CFI = 0.97; TLI = 0.96; RMSEA = 0.02)

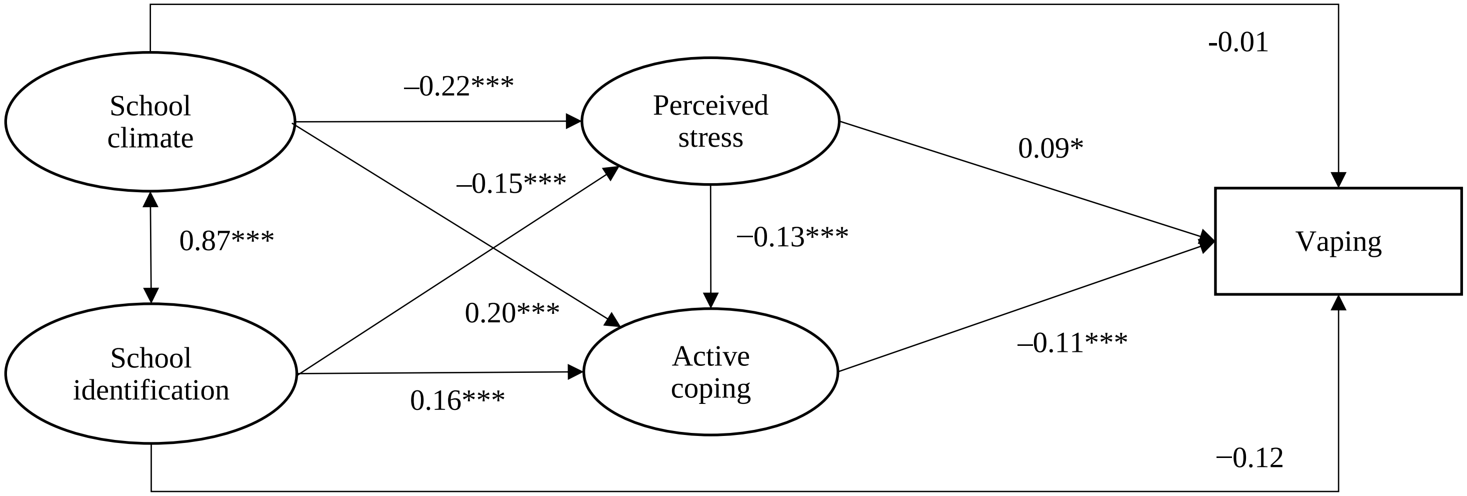

Figure S3 Structural equation modeling (**, p<0.01; ***, p<0.001. Rectangles represent observed variables, ovals represent latent variables, arrows represent the directions of the associations, and straight lines with coefficients represent the standardized path coefficients. The model was adjusted for background factors, including age, school types, gender, whether living in the studied city constantly, whether living with both parents, and father's and mother's educational level. Model fit indices: CFI = 0.97; TLI = 0.96; RMSEA = 0.02)

© 2024 Yu Y. et al.
